# Lessons Learned from the Impact of HIV Status Disclosure to Children after First-Line Antiretroviral Treatment Failure in Kinshasa, DR Congo

**DOI:** 10.3390/children9121955

**Published:** 2022-12-13

**Authors:** Faustin Nd. Kitetele, Gilbert M. Lelo, Cathy E. Akele, Patricia V. M. Lelo, Loukia Aketi, Eric M. Mafuta, Thorkild Tylleskär, Espérance Kashala-Abotnes

**Affiliations:** 1 Department of Infectious Diseases, Kalembelembe Pediatric Hospital, Kinshasa 012, Democratic Republic of the Congo; 2 Centre for International Health (CIH), Faculty of Medicine, University of Bergen, 5020 Bergen, Norway; 3 Centre Neuro-Psycho-Pathologique de Kinshasa (CNPP), Faculty of Medicine, University of Kinshasa, Kinshasa 012, Democratic Republic of the Congo; 4 Pediatric Department, Faculty of Medicine, University of Kinshasa, Kinshasa 012, Democratic Republic of the Congo; 5 Kinshasa School of Public Health, Faculty of Medicine, University of Kinshasa, Kinshasa 012, Democratic Republic of the Congo

**Keywords:** HIV, antiretroviral therapy failure, pediatrics, depression, disclosure, compliance

## Abstract

HIV status disclosure to children remains a challenge in sub-Saharan Africa. For sociocultural reasons, parents often delay disclosure with subsequent risks to treatment compliance and the child’s psychological well-being. This article assesses the effects of HIV disclosure on second-line ART compliance after first-line failure. We conducted a retrospective study of 52 HIV-positive children at Kalembelembe Pediatric Hospital in Kinshasa who were unaware of their HIV status and had failed to respond to the first-line ART. Before starting second-line ART, some parents agreed to disclosure. All children were followed before and during the second-line ART. Conventional usual descriptive statistics were used. For analysis, the children were divided into two groups: disclosed to (*n* = 39) and not disclosed to (*n* = 13). Before starting the second-line ART, there was no difference in CD4 count between the two groups (*p* = 0.28). At the end of the first year of second-line ART, the difference was statistically significant between the two groups with regard to CD4% (*p* < 0.001) and deaths (*p* = 0.001). The children disclosed to also reported fewer depressive symptoms post-disclosure and had three times fewer clinic visits. HIV status disclosure to children is an important determinant of ART compliance and a child’s psychological well-being.

## 1. Introduction

Africa remains the continent most heavily affected by HIV/AIDS, accounting for 68% of people living with HIV (PLHIV) in 2021 [1]. Young people are at the centre of the global epidemic [2]. Adolescents or youth who are living with HIV may have multiple developmental, cognitive, social, contextual, and environmental challenges that impact their identification, linkage, and engagement with care [3]. Studies indicate that if children living with HIV know about their HIV status, they are more likely to adhere to antiretroviral treatment (ART), and this may have a positive influence on their quality of life [4,5].

A systematic review of studies on HIV status disclosure to children in sub-Saharan Africa showed that the prevalence of HIV disclosure ranged from as low as 9% to 72% [6]. The decision to disclose or not was justified by different factors related to the child, caregivers, and health institution. Evidence from these studies suggests that disclosure may affect the child’s adherence to medication, treatment outcome, and behaviours related to the risk of transmission of HIV to others [4,7,8].

According to the 2021 annual report of the AIDS National Program, the prevalence of HIV in the Democratic Republic of Congo (DRC) was 1.2% of the general population, and the number of PLHIV was about 506,000, of which 72,000 were children aged under 15. Of these children, only 33% were on ART, and very few had their HIV status disclosed to them.

In 2007, a four-stage HIV disclosure process was created and implemented in the DRC and later described in the “WHO guideline on HIV disclosure counselling for children up to 12 years of age” in 2011 [9]. However, the DRC has not yet assessed the effects of HIV disclosure on children.

The main objective of this study was to assess the effects of HIV disclosure on compliance with the second-line antiretroviral therapy (ART2) after first-line ART (ART1) failure, measured by (a) the evolution of CD4+ T-cell count/percentage and viral load before and after disclosure, (b) deaths, and (c) depressive symptoms.

## 2. Methods and Design

### 2.1. Study Design and Setting

A retrospective hospital-based study was conducted using anonymous information from patient records followed at Kalembelembe Pediatric Hospital in Kinshasa, DRC.

Kalembelembe Pediatric Hospital (KLLPH) is a public health facility specializing in managing children and adolescents with infectious diseases such as HIV and was the first centre in the country (in 2002) to provide ART and follow-up to HIV-exposed children.

### 2.2. Participants, Data Collection, and Procedures

This study included children living with HIV aged from 6 to 17 years at KLLPH between December 2004 and November 2008, whose HIV-positive status was undisclosed to them and who were non-responsive to first-line ART. Those with unknown HIV disclosure status were excluded. All medical records of the study population containing relevant information on ART and disclosure were listed.

For children who were failing on first-line ART and where the parents had initially refused disclosure, a meeting was held with parents to try to convince them to approve disclosure before initiating the second-line ART. The parents of 39 children consented to the disclosure, and others did not (13 children). Eventually—after around two years—these children were also disclosed to.

During the study period, the process of HIV status disclosure to children was handled by healthcare workers (HCWs). The full disclosure was done by caregivers or HCWs, involving or not “peer educators” [10], after consent and approval from caregivers.

After disclosure, the level of acceptance was assessed, and the child was screened for possible symptoms of depression. After disclosure, the patient was encouraged to join a children’s self-support group (SSG) [10].

Data were collected between October and December 2017 on a structured data extraction form, which was specially designed to collect information from the medical records of the patients and the database of KLLPH. Information on the disclosure of HIV status procedure, medical history, socio-demographic characteristics, the clinical stage, the CD4 count/percentage and their evolution on first-line ART, and the viral load (VL) were collected. The health status of the children was evaluated through a review of the chart for comments regarding the clinical examination and comments on the mental health of the child.

The clinical stage of HIV was recorded in accordance with the WHO classification [11]. In addition, the CD4 count/percentage was evaluated at baseline (initiation of first-line ART), during follow-up visits at 12 and 24 months, and after the failure of first-line ART was confirmed.

Patients who had failed first-line ART were started on second-line ART. The CD4 count/percentage was evaluated at 6, 12, 24, 36, and 48 months. The VL data were assessed to confirm ART1 failure and again after 4 years on ART2 for long-term follow-up. Patient records with missing data on one or more of the covariates were censored from the multivariable analysis. All biological analyses were conducted at the National Laboratory of Reference for HIV-AIDS in Kinshasa, using the FACSCalibur, (Becton Dickinson Immunocytometry Systems, San Jose, CA, USA) for CD4 count and the Abbott m 2000rt Real-time HIV-1 assay (Abbott Laboratories, Chicago, IL, USA) for VL.

### 2.3. Sample Size

A total of 564 patients aged 6 to 17 years old were followed up with and treated on first-line ART from 2004 to 2008 at KLLPH. Of them, 52 (9.2%) were non-responsive to first-line ART and constituted our study population.

### 2.4. Outcome Variables

The main outcome variables were the effects of disclosure or non-disclosure of the child’s HIV status on compliance to second-line therapy evaluated by CD4 count/percentage, viral load, survival, and depressive symptoms.

### 2.5. Operational Definitions

Disclosure: The process by which a child is incrementally made aware of their HIV status in an age-appropriate manner [9].

Full disclosure: When the caregiver or HCW announces to a child that they are infected and uses the term HIV in their explanation during the disclosure process.

Partial disclosure: When the child is informed that they have chronic health problems but not told specifically that the disease is HIV.

Non-disclosure: When the caregiver or HCW reported telling the child nothing about their illness.

Loss to follow-up: Children not attending the scheduled follow-up visits for three consecutive visits, and all attempts to retrieve the patient for follow-up failed without unscheduled visits noted during this period [12,13].

Immunologic failure: Fall of CD4 count to below baseline in the absence of concurrent infections, a fall of more than 50% from the peak value, or persistent CD4 below 100 cells/mm^3^ while on treatment [14].

Immunologic recovery: An increase in CD4 count of ≥ 25% from pre-therapy baseline levels [13,15].

Virologic suppression: Plasma HIV-1 RNA < 1000 copies/mL after 12 months of ART [13].

Virologic failure: Plasma HIV-RNA over 1000 copies/mL after two consecutive viral load measurements in 3 months, with adherence support following the first viral load test [14,16].

Depression: According to the American Psychological Association, depression or major depressive disorder is a common and serious medical illness that negatively affects how you feel, the way you think, and how you act.

Evaluation of depressive symptoms after HIV disclosure:

The Hamilton Depression Rating Scale (HDRS) [17] was used to detect the presence of depressive symptoms. It provides information on the presence of symptoms over time and on the recovery. The scale is one of the most widely used to evaluate the presence and severity of depressive symptoms. The HDRS is a 24-item scale comprising 7 items on anxiety and 17 items on somatic symptoms [18]. The total score is obtained by the addition of the first seventeen items, and the remainder provides additional clinical information.

### 2.6. Data Analysis

Data were captured on Microsoft Excel, and the IBM^®^ SPSS^®^ Statistics for Microsoft Windows version 23.0 (Armonk, NY, USA) and Stata 14 (College Station, TX, USA) were used for statistical analysis.

The categorical variables were presented in the form of proportions with a 95% confidence interval. The average and standard deviation were calculated for continuous variables with a symmetric distribution. The median, with extremes, was calculated for continuous variables with the asymmetric distribution.

The Kolmogorov-Smirnov and Shapiro-Wilk tests, as well as the -Q plots, were used to test and appreciate the distribution of the numerical variables. Repeated-measures ANOVA was used to compare the means of different CD4 groups. Variables with skewed distribution were standardized to a standard normal distribution of a mean 0 and standard deviation of 1 to fulfil the condition of the normality of the variables to be introduced into the repeated-measures ANOVA test model. *p*-value < 0.05 was considered as statistically significant for treatment failure.

### 2.7. Ethics

All parents of the patients signed an informed consent for participation in the HIV care program before enrolment. Children older than 12 years of age provided their assent.

Ethical approval was obtained from the DRC National Health Ethics Committee at the Ministry of Health (code: 040/CNES/BN/PMMF/2017, 21 June 2017) and the Norwegian Regional Committee for Medical and Health Research Ethics (code: 2018/1525/REK Vest).

## 3. Results

### 3.1. Sample Description

**Figure 1:**
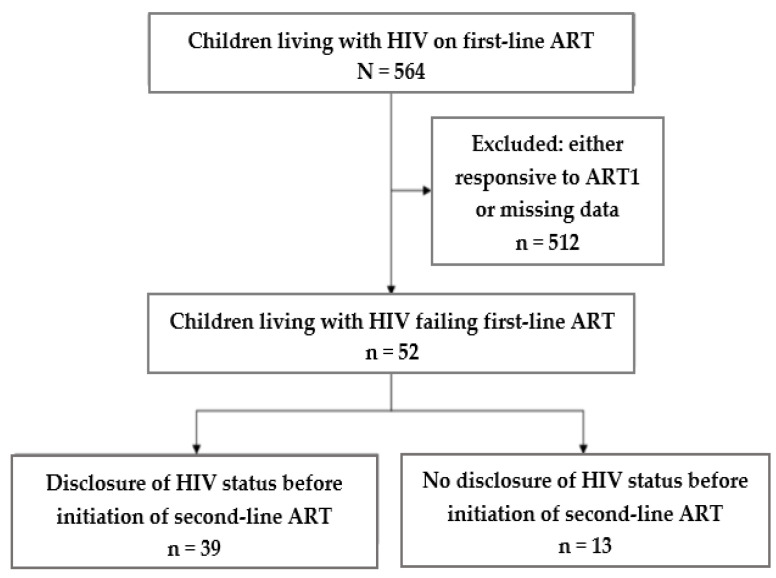
Flow diagram showing the children and adolescents on ART at KLLPH, DRC, between 2004 and 2008 and the group who failed on ART1.

A total of 564 patients aged 6 to 17 years old were followed up with and treated under ART1 from 2004 to 2008 at KLLPH. After applying the inclusion and exclusion criteria, 90.8% were either responsive to ART1 or the patient records had missing data and were excluded from the analysis. The remaining 9.2% were non-responsive to ART1 and constituted our study population (Figure 1).

### 3.2. Socio-Demographic Characteristics

Of the 52 patients enrolled in the study, 30 (58%) were boys. The mean age was 10.8 years (standard deviation, SD: 4.1), with an average treatment duration of 3.4 years (SD: 1.2). In the analysis, the children were divided into two groups: the group whose parents accepted the subsequent disclosure (*n* = 39, 75%) and the group whose parents refused it (*n* = 13, 25%) (Table 1).

There was no statistically significant difference in parental status between the two groups (*p* = 0.12), Table 1. The majority of children who failed first-line ART were orphans (76.9%).

### 3.3. First-Line ART Characteristics

At enrolment, 15 (29%) of the 52 children were classified in the WHO clinical stage 1 or 2, and 37 (71%) were in stage 3 or 4. All had previously been provided first-line ART in accordance with the national guidelines. The median CD4% following initiation of first-line therapy increased from 5% (95% CI 2–10%) to 13% (95% CI 7.5–20.5%) after one year and then to 11% (95% CI 5–17.5%) after two years. None of the 52 children had been disclosed to or benefited from any disclosure process. After 3.3 years, the average CD4% fell to 5.6% (range: 0 to 31%), and the average VL was high at 255,000 (range: 2100 to 1,760,000) RNA copies/mL, thus fulfilling the criteria for immunological and virological failure, and therefore considered as a first-line ART treatment failure.

Before initiating second-line ART, 39 (75%) parents/guardians accepted to immediately start the disclosure process for partial or full disclosure of HIV status. Meanwhile, 13 (25%) parents/guardians refused to disclose HIV status to their children.

Among the 39 children whose parents/guardians accepted the disclosure, 23 (59%) aged over 12 years were disclosed to within 2 to 4 weeks, and 16 (41%), aged between 6 and 11 years, started the disclosure process in line with the protocol in place.

### 3.4. Follow-Up at 1 and 4 Years after Initiating Second-Line ART

Before initiating second-line ART, there was no significant difference in CD4% between the children disclosed to vs. children not disclosed to, see Table 2. Over the first year, the CD4% improved in the group that was disclosed to but not in the other group. The difference in CD4%, therefore, became significantly different between the two groups (14.6 vs. 6.8%, *p* = 0.005) at the end of the first year on second-line ART (Table 2).

Among the children that were disclosed to, there was one death (1/39, 2.6%) and in the group not disclosed to almost half died (6/13, 46.2%) (*p* = 0.001) in the first year following the initiation of second-line therapy.

### 3.5. Comparison of CD4% Progression from First-Line ART to Second-Line ART between Children Disclosed to vs. Children Not Disclosed to

**Figure 2:**
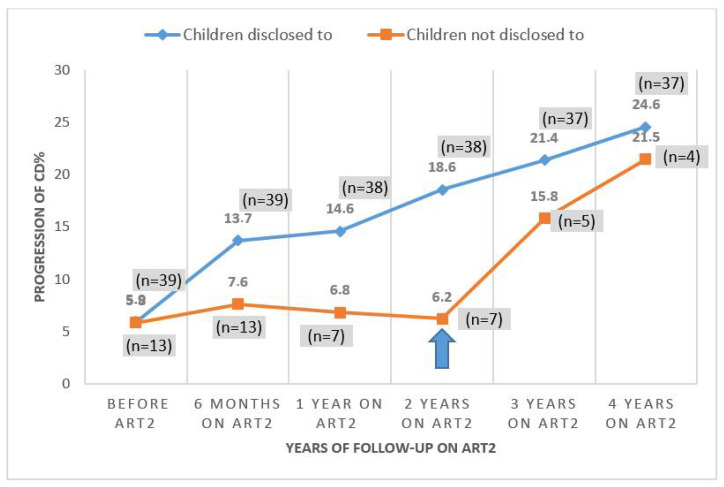
Evolution of the CD4% in the two groups (children immediately disclosed to (blue) vs. children not immediately disclosed to (orange) then disclosed to after 2 years (blue arrow)) during 4 years on second-line ART.

However, the CD4% improved dramatically in the group disclosed to when they started second-line ART in sharp contrast to the curve of children not disclosed to, which only shows a slight increase, then stagnates, and later rapidly re-increased when finally—after 2 years—they were disclosed to (Figure 2).

### 3.6. Comparison of the Evolution of the VL 4 Years Later between the Disclosed vs. Late Disclosed Groups

The average VL before second-line ART was 255,000 RNA copies/mL (range 2100–1,760,000) for the children immediately disclosed to and 144,000 RNA copies/mL (range 4000–950,000) for the children disclosed to late. After four years on second-line ART, 71% (27/38) of the children immediately disclosed to had a suppressed VL versus 50% (2/4) of the children that were disclosed to late.

### 3.7. Evaluation of Post-Disclosure Depression

#### 3.7.1. Evaluation of Post-Disclosure Depression Symptoms in 32 Children Having Been Disclosed to Before Starting Second-Line ART

Of the 32 children disclosed to early, depressive symptoms were documented in 23 (71.9%) of them. At the end of the first month on second-line ART, severe or very severe depression symptoms were present in 13 out of 23 children (56.5%) who had had their HIV status fully disclosed to them. Three months later, after integration into a self-support group, 1 child presented severe symptoms, and 22 children had mild, or no symptoms. At 6 months, one child presented severe symptoms.

#### 3.7.2. Monitoring of Post-Disclosure Depression Symptoms of Seven Children on Second-Line ART Initiated without Prior Disclosure

In the group not disclosed to before initiating ART2, the ones still alive were eventually disclosed to. The average age for disclosure was 15 years, and 6 out of 7 children attended an SSG. After disclosure, three children presented symptoms of depression, and two amongst them were lost to follow-up. The third had refused to take ARVs and was eventually lost to follow-up.

## 4. Discussion

This study is the first in DR Congo to assess the effects of HIV disclosure on ART compliance among children after first-line ART failure. Our main finding is that adolescents failing on first-line ART and moving to second-line ART do much better if they are disclosed to before the shift. This is in line with previous studies that have shown that early and full disclosure is strongly associated with improved adherence amongst ART-initiated adolescents [13,15,19,20,21]. Poor adherence to treatment is one, if not the main, cause of treatment failure and most often occurs due to non-disclosure or lack of knowledge about the disease [22]. Disclosure is an important component of the comprehensive management of children living with HIV. However, disclosing rates tend to be low, especially in low-income countries. For some parents, the most frequently stated reason for non-disclosure was the belief that the child was too young to understand his illness [23,24]. Others have found that 11-year-old boys were seven times more likely than girls to have treatment failure [25,26].

In our study, a 9.2% failure rate was recorded in patients with a low CD4% (on average 5.5%). These data are consistent with most studies in Africa where children are often diagnosed with advanced diseases. Previous studies suggest a relationship between the advanced clinical stage at therapy initiation and treatment failure [27,28,29,30,31].

In our study, most registered deaths happened in the group of children whose parents had refused disclosure. Therapeutic failure and death are very often seen as part of the normal course of the disease in HIV-infected adolescents. Many families are reluctant to discuss the nature of the illness with their infected child or adolescent. Delaying disclosure of HIV infection may potentially result in negative consequences. Lack of disclosure may impair treatment understanding and participation and increase psychological and behavioural problems [10,32,33,34,35]. For the health system, non-disclosure of HIV status and treatment failures increase providers’ workload and the direct and indirect costs associated with the management of opportunistic infections, repeated sessions of adherence counselling, multiple appointments, and even hospitalizations [14].

The lessons learned are that children disclosed to had significantly better ART adherence and psychological well-being compared to children not disclosed to.

Early and full disclosure is strongly associated with improved adherence in our study.

In fact, the disclosure provides the adolescent with appropriate and truthful explanations of HIV, dissipates concerns or fears, clarifies misconceptions about HIV, and prepares the child or adolescent to deal with stigma. This allows them to accept their diagnosis, adhere to ART, and be more engaged with HIV care, resulting in better HIV treatment outcomes, higher self-esteem, and improved long-term mental health outcomes compared with children and adolescents not disclosed to [10,36,37].

However, full disclosure, even with good preparation, may be followed by mental health reactions such as sadness, anger, rebellion, fear, perceived stigma, internalized stigma, perceived shortened future, and fear of death [10,36].

Therefore, it is important for caregivers and HCWs to change the practice of non-disclosure of HIV status to children and to incrementally fully disclose, taking into account their cognitive skills and emotional maturity.

## 5. Strengths and Limitations of the Study

This study is the first in DR Congo that assesses the effects of HIV disclosure on children after first-line ART failure. However, our findings require careful interpretation due to the limitations, which include: (1) the small sample size and the single study site; (2) the retrospective nature of the work that possibly led to missing data and bias; and (3) other factors that may explain poor adherence or therapeutic failure, such as primary resistance to first-line ART, were not considered.

However, despite the fact that the information collected in the present study is dated, we believe that our findings are of great importance in this context as it provides useful information on current challenges faced in the process of HIV status disclosure among adolescents and its impact on monitoring.

## 6. Conclusions

HIV disclosure to children living with HIV is important as disclosure is likely to improve both ART compliance and the overall well-being of the children.

## Figures and Tables

**Table 1:** Family situation of the study children failing first-line ART.

	Children Disclosed to (*N* = 39)		Children Not Disclosed to (*N* = 13)		Total (*N* = 52)	
	*n*	(%)	*N*	(%)	*n*	(%)
Family situation of the child:						
Double orphan	15	(38.5)	4	(30.8)	19	(36.5)
Paternal orphan	8	(20.5)	0	(0.0)	8	(15.4)
Maternal orphan	7	(17.9)	6	(46.1)	13	(25.0)
Both parents alive	9	(23.1)	3	(23.1)	12	(23.1)

**Table 2:** CD4% at different time points between children disclosed to vs. children not disclosed to.

	Children Disclosed to (*N* = 39)		Children Not Disclosed to (*N* = 13)		*p*-Value
	Median	Range	Median	Range	
CD4% before ART1	5.7	0–18	7.8	0–19	0.22
CD4% before ART2	5.9	0–21	5.8	0–20	0.59
CD4% 1 year after ART2	14.6	SD 7.4	6.8	SD 6.8	0.005

## Data Availability

Data supporting reported results can be requested from the corresponding author upon reasonable request.
